# Supplementary material for: Safety of Dual Orexin Receptor Antagonist Daridorexant: A Disproportionality Analysis of Publicly Available FAERS Data
Source: Pharmaceuticals (Basel). 2024 Mar 6;17(3):342. doi: 10.3390/ph17030342 (PMC10974491; doi:10.3390/ph17030342)
Supplement: Supplementary file 1 [file pharmaceuticals-17-00342-s001.zip › pharmaceuticals-2898242-supplementary.pdf]

Supplementary Material

Safety of Dual Orexin Receptor Antagonist Daridorexant: A Disproportionality Analysis of Publicly Available FAERS Data

Table S1: Adverse Drug Reactions Observed in Daridorexant and other Dual Orexin Receptor Antagonists related Individual Case Safety Reports regrouped by High Level Group Terms

High Level Group Terms (HLGTs)	Daridorexant ICSRs[a] n (%)	Other DORA ICSRs[a] n (%)
Sleep disorders and disturbances	326 (38.6)	2264 (25.2)
Therapeutic and nontherapeutic effects (excl toxicity)	311 (36.8)	3164 (35.2)
General system disorders NEC	194 (23)	1088 (12.1)
Neurological disorders NEC	114 (13.5)	1181 (13.1)
Medication errors and other product use errors and issues	102 (12.1)	891 (9.9)
Headaches	69 (8.2)	454 (5.1)
Product quality, supply, distribution, manufacturing and quality system issues	69 (8.2)	187 (2.1)
Disturbances in thinking and perception	44 (5.2)	410 (4.6)
Gastrointestinal signs and symptoms	41 (4.9)	346 (3.9)
Anxiety disorders and symptoms	40 (4.7)	413 (4.6)
Epidermal and dermal conditions	34 (4)	179 (2)
Sleep disturbances (incl subtypes)	32 (3.8)	369 (4.1)
Allergic conditions	30 (3.6)	45 (0.5)
Movement disorders (incl parkinsonism)	26 (3.1)	238 (2.6)
Depressed mood disorders and disturbances	25 (3)	132 (1.5)
Overdoses and underdoses NEC	23 (2.7)	273 (3)
Respiratory disorders NEC	22 (2.6)	175 (1.9)
Skin appendage conditions	21 (2.5)	90 (1)

Gastrointestinal motility and defaecation conditions	21 (2.5)	137 (1.5)
Cardiac disorders, signs and symptoms NEC	16 (1.9)	106 (1.2)
Suicidal and self-injurious behaviours NEC	16 (1.9)	166 (1.8)
Salivary gland conditions	14 (1.7)	68 (0.8)
Muscle disorders	14 (1.7)	150 (1.7)
Mood disorders and disturbances NEC	11 (1.3)	117 (1.3)
Cardiac and vascular investigations (excl enzyme tests)	11 (1.3)	95 (1.1)
Deliria (incl confusion)	11 (1.3)	191 (2.1)
Changes in physical activity	10 (1.2)	57 (0.6)
Lifestyle issues	10 (1.2)	32 (0.4)
Off label uses and intentional product misuses/use issues	9 (1.1)	105 (1.2)
Mental impairment disorders	9 (1.1)	189 (2.1)
Oral soft tissue conditions	9 (1.1)	31 (0.3)
Fatal outcomes	8 (0.9)	43 (0.5)
Urinary tract signs and symptoms	8 (0.9)	88 (1)
Inner ear and VIIIth cranial nerve disorders	7 (0.8)	48 (0.5)
Injuries NEC	7 (0.8)	162 (1.8)
Respiratory tract signs and symptoms	7 (0.8)	50 (0.6)
Musculoskeletal and connective tissue disorders NEC	7 (0.8)	90 (1)
Viral infectious disorders	6 (0.7)	26 (0.3)
Eye disorders NEC	6 (0.7)	27 (0.3)
Psychiatric and behavioural symptoms NEC	6 (0.7)	58 (0.6)
Angioedema and urticaria	6 (0.7)	22 (0.2)
Appetite and general nutritional disorders	5 (0.6)	52 (0.6)
Cardiac arrhythmias	5 (0.6)	71 (0.8)
Physical examination and organ system status topics	5 (0.6)	55 (0.6)
Infections - pathogen unspecified	5 (0.6)	74 (0.8)
Vision disorders	4 (0.5)	43 (0.5)

Vascular disorders NEC	3 (0.4)	18 (0.2)
Sexual dysfunctions, disturbances and gender identity disorders	3 (0.4)	9 (0.1)
Personality disorders and disturbances in behaviour	3 (0.4)	45 (0.5)
Upper respiratory tract disorders (excl infections)	3 (0.4)	28 (0.3)
Hepatic and hepatobiliary disorders	2 (0.2)	42 (0.5)
Tongue conditions	2 (0.2)	13 (0.1)
Cognitive and attention disorders and disturbances	2 (0.2)	7 (0.1)
Therapeutic procedures and supportive care NEC	2 (0.2)	19 (0.2)
Seizures (incl subtypes)	2 (0.2)	68 (0.8)
Bone disorders (excl congenital and fractures)	1 (0.1)	9 (0.1)
Bronchial disorders (excl neoplasms)	1 (0.1)	8 (0.1)
Cytogenetic investigations and genetic analyses	1 (0.1)	1 (0)
Central nervous system vascular disorders	1 (0.1)	37 (0.4)
Aural disorders NEC	1 (0.1)	1 (0)
Decreased and nonspecific blood pressure disorders and shock	1 (0.1)	16 (0.2)
Cranial nerve disorders (excl neoplasms)	1 (0.1)	7 (0.1)
Ocular infections, irritations and inflammations	1 (0.1)	12 (0.1)
Urolithiases	1 (0.1)	3 (0)
Toxicology and therapeutic drug monitoring	1 (0.1)	20 (0.2)
Skin vascular abnormalities	1 (0.1)	5 (0.1)
Renal disorders (excl nephropathies)	1 (0.1)	27 (0.3)
Psychiatric disorders NEC	1 (0.1)	56 (0.6)
Metabolic, nutritional and blood gas investigations	1 (0.1)	26 (0.3)
Ocular sensory symptoms NEC	1 (0.1)	7 (0.1)
Dental and gingival conditions	1 (0.1)	4 (0)
Nervous system, skull and spine therapeutic procedures	1 (0.1)	1 (0)
Vulvovaginal disorders (excl infections and inflammations)	1 (0.1)	2 (0)
Middle ear disorders (excl congenital)	1 (0.1)	1 (0)

Joint disorders	1 (0.1)	37 (0.4)
Hearing disorders	1 (0.1)	11 (0.1)
Gastrointestinal haemorrhages NEC	1 (0.1)	8 (0.1)
Prostatic disorders (excl infections and inflammations)	1 (0.1)	5 (0.1)

[a]*ICSR: Individual Case Safety Report*

Table S2: Adverse Drug Reactions Observed in Daridorexant and other Dual Orexin Receptor Antagonists related Individual Case Safety Reports by High Level Group Terms and Preferred Terms

High Level Group Terms (HLGTs)	Preferred Terms (PTs)	Daridorexant ICSRs[a] N (%)	Other DORA ICSRs[a] N (%)
Sleep disorders and disturbances	Nightmare	146 (17.3)	729 (8.1)
	Insomnia	80 (9.5)	449 (5)
	Abnormal dreams	64 (7.6)	588 (6.5)
	Somnambulism	18 (2.1)	127 (1.4)
	Middle insomnia	15 (1.8)	159 (1.8)
	Poor quality sleep	10 (1.2)	187 (2.1)
	Sleep terror	9 (1.1)	52 (0.6)
	Sleep-related eating disorder	4 (0.5)	16 (0.2)
	Initial insomnia	4 (0.5)	69 (0.8)
	Hypnagogic hallucination	3 (0.4)	8 (0.1)
	Sleep talking	2 (0.2)	10 (0.1)
	Parasomnia	2 (0.2)	5 (0.1)
	Abnormal sleep-related event	2 (0.2)	32 (0.4)
	Sleep disorder	1 (0.1)	170 (1.9)
Therapeutic and nontherapeutic effects (excl toxicity)	Drug ineffective	233 (27.6)	2699 (30)
	Therapeutic product effect incomplete	33 (3.9)	167 (1.9)
	Therapeutic product effect delayed	18 (2.1)	51 (0.6)
	Therapeutic product effect variable	8 (0.9)	10 (0.1)
	Therapeutic product effect decreased	8 (0.9)	73 (0.8)
	Drug intolerance	5 (0.6)	14 (0.2)
	Drug interaction	4 (0.5)	41 (0.5)
	Therapeutic response unexpected	4 (0.5)	35 (0.4)
	Drug effect less than expected	1 (0.1)	9 (0.1)

	Treatment failure	1 (0.1)	6 (0.1)
	Drug withdrawal syndrome	1 (0.1)	4 (0)
	Therapeutic response delayed	1 (0.1)	14 (0.2)
General system disorders NEC	Feeling abnormal	64 (7.6)	395 (4.4)
	Fatigue	45 (5.3)	209 (2.3)
	Hangover	20 (2.4)	63 (0.7)
	Illness	17 (2)	10 (0.1)
	Malaise	12 (1.4)	116 (1.3)
	Asthenia	6 (0.7)	52 (0.6)
	Feeling jittery	6 (0.7)	51 (0.6)
	Energy increased	5 (0.6)	8 (0.1)
	Crying	5 (0.6)	19 (0.2)
	Concomitant disease aggravated	4 (0.5)	-
	Chest pain	4 (0.5)	30 (0.3)
	Pain	4 (0.5)	51 (0.6)
	Sluggishness	4 (0.5)	13 (0.1)
	Discomfort	3 (0.4)	11 (0.1)
	Condition aggravated	3 (0.4)	16 (0.2)
	Screaming	3 (0.4)	12 (0.1)
	Swelling face	3 (0.4)	11 (0.1)
	Chills	2 (0.2)	19 (0.2)
	Chest discomfort	2 (0.2)	21 (0.2)
	Feeling cold	2 (0.2)	7 (0.1)
	Peripheral swelling	2 (0.2)	7 (0.1)
	Gait disturbance	2 (0.2)	35 (0.4)
	Gait inability	2 (0.2)	4 (0)
	Performance status decreased	1 (0.1)	-
	Influenza like illness	1 (0.1)	7 (0.1)

	Swelling	1 (0.1)	3 (0)
	Localised oedema	1 (0.1)	(0)
	Face oedema	1 (0.1)	1 (0)
	Feeling drunk	1 (0.1)	16 (0.2)
Neurological disorders NEC	Somnolence	55 (6.5)	600 (6.7)
	Dizziness	29 (3.4)	213 (2.4)
	Lethargy	8 (0.9)	25 (0.3)
	Restless legs syndrome	7 (0.8)	34 (0.4)
	Hypoaesthesia	6 (0.7)	35 (0.4)
	Sedation	5 (0.6)	36 (0.4)
	Paraesthesia	4 (0.5)	34 (0.4)
	Speech disorder	2 (0.2)	27 (0.3)
	Dysgeusia	2 (0.2)	24 (0.3)
	Balance disorder	1 (0.1)	23 (0.3)
	Loss of consciousness	1 (0.1)	48 (0.5)
	Ageusia	1 (0.1)	2 (0)
	Head discomfort	1 (0.1)	26 (0.3)
	Myoclonus	1 (0.1)	5 (0.1)
	Neuralgia	1 (0.1)	-
	Burning sensation	1 (0.1)	10 (0.1)
	Aphasia	1 (0.1)	15 (0.2)
Medication errors and other product use errors and issues	Inappropriate schedule of product administration	48 (5.7)	82 (0.9)
	Wrong technique in product usage process	35 (4.1)	210 (2.3)
	Product prescribing error	8 (0.9)	15 (0.2)
	Product use issue	4 (0.5)	108 (1.2)
	Product prescribing issue	4 (0.5)	13 (0.1)

	Product dispensing error	3 (0.4)	2 (0)
	Contraindicated product administered	2 (0.2)	9 (0.1)
	Intercepted product dispensing error	1 (0.1)	1 (0)
	Intercepted product prescribing error	1 (0.1)	1 (0)
	Product storage error	1 (0.1)	22 (0.2)
	Incorrect dose administered	1 (0.1)	39 (0.4)
Headaches	Headache	59 (7)	419 (4.7)
	Migraine	8 (0.9)	38 (0.4)
	Migraine with aura	2 (0.2)	1 (0)
Product quality, supply, distribution, manufacturing and quality system issues	Product availability issue	51 (6)	18 (0.2)
	Product packaging difficult to open	7 (0.8)	13 (0.1)
	Product taste abnormal	2 (0.2)	2 (0)
	Product physical issue	2 (0.2)	3 (0)
	Product container seal issue	2 (0.2)	-
	Product container issue	2 (0.2)	2 (0)
	Product shape issue	1 (0.1)	1 (0)
	Product after taste	1 (0.1)	-
	Product packaging issue	1 (0.1)	10 (0.1)
	Product quality issue	1 (0.1)	67 (0.7)
	Product colour issue	1 (0.1)	6 (0.1)
Disturbances in thinking and perception	Hallucination	30 (3.6)	317 (3.5)
	Tachyphrenia	5 (0.6)	14 (0.2)
	Hallucination, visual	3 (0.4)	19 (0.2)
	Hallucination, auditory	2 (0.2)	25 (0.3)
	Autoscopy	2 (0.2)	6 (0.1)
	Bradyphrenia	1 (0.1)	1 (0)
	Thinking abnormal	1 (0.1)	31 (0.3)
	Delusion	1 (0.1)	6 (0.1)

Gastrointestinal signs and symptoms	Nausea	23 (2.7)	197 (2.2)
	Abdominal discomfort	8 (0.9)	43 (0.5)
	Vomiting	7 (0.8)	65 (0.7)
	Abdominal pain upper	5 (0.6)	24 (0.3)
	Abdominal pain	1 (0.1)	20 (0.2)
	Abdominal distension	1 (0.1)	7 (0.1)
	Dyspepsia	1 (0.1)	18 (0.2)
	Dysphagia	1 (0.1)	22 (0.2)
Anxiety disorders and symptoms	Agitation	16 (1.9)	98 (1.1)
	Anxiety	14 (1.7)	203 (2.3)
	Nervousness	5 (0.6)	43 (0.5)
	Panic attack	3 (0.4)	29 (0.3)
	Fear	1 (0.1)	40 (0.4)
	Phobia of driving	1 (0.1)	1 (0)
	Stress	1 (0.1)	16 (0.2)
Epidermal and dermal conditions	Pruritus	15 (1.8)	63 (0.7)
	Rash	15 (1.8)	68 (0.8)
	Erythema	4 (0.5)	14 (0.2)
	Skin irritation	1 (0.1)	-
	Skin odour abnormal	1 (0.1)	1 (0)
	Rash papular	1 (0.1)	1 (0)
	Psoriasis	1 (0.1)	2 (0)
	Dermatitis contact	1 (0.1)	-
	Rash erythematous	1 (0.1)	4 (0)
Sleep disturbances (incl subtypes)	Sleep paralysis	21 (2.5)	278 (3.1)
	Hypersomnia	10 (1.2)	38 (0.4)
	Narcolepsy	1 (0.1)	8 (0.1)
Allergic conditions	Hypersensitivity	29 (3.4)	11 (0.1)

	Drug hypersensitivity	1 (0.1)	22 (0.2)
Movement disorders (incl parkinsonism)	Tremor	15 (1.8)	75 (0.8)
	Dyskinesia	5 (0.6)	17 (0.2)
	Psychomotor hyperactivity	3 (0.4)	27 (0.3)
	Bradykinesia	1 (0.1)	-
	Paralysis	1 (0.1)	43 (0.5)
	Resting tremor	1 (0.1)	-
	Freezing phenomenon	1 (0.1)	-
Depressed mood disorders and disturbances	Depression	22 (2.6)	101 (1.1)
	Depressed mood	3 (0.4)	21 (0.2)
	Depression suicidal	2 (0.2)	1 (0)
Overdoses and underdoses NEC	Intentional overdose	17 (2)	56 (0.6)
	Prescribed overdose	3 (0.4)	21 (0.2)
	Overdose	3 (0.4)	133 (1.5)
	Prescribed underdose	1 (0.1)	7 (0.1)
Respiratory disorders NEC	Dyspnoea	20 (2.4)	102 (1.1)
	Respiratory tract congestion	1 (0.1)	-
	Cough	1 (0.1)	28 (0.3)
Gastrointestinal motility and defaecation conditions	Diarrhoea	15 (1.8)	98 (1.1)
	Constipation	4 (0.5)	23 (0.3)
	Gastrooesophageal reflux disease	1 (0.1)	13 (0.1)
	Diarrhoea haemorrhagic	1 (0.1)	-
	Irritable bowel syndrome	1 (0.1)	-
Skin appendage conditions	Night sweats	11 (1.3)	30 (0.3)
	Hyperhidrosis	8 (0.9)	48 (0.5)
	Alopecia	2 (0.2)	1 (0)
Cardiac disorders, signs and symptoms NEC	Palpitations	15 (1.8)	99 (1.1)
	Cardiac discomfort	1 (0.1)	-

Suicidal and self-injurious behaviours NEC	Suicidal ideation	13 (1.5)	77 (0.9)
	Suicide attempt	2 (0.2)	40 (0.4)
	Completed suicide	1 (0.1)	35 (0.4)
Muscle disorders	Muscle spasms	5 (0.6)	34 (0.4)
	Muscular weakness	3 (0.4)	53 (0.6)
	Muscle twitching	3 (0.4)	12 (0.1)
	Myalgia	3 (0.4)	32 (0.4)
	Muscle rigidity	1 (0.1)	5 (0.1)
Salivary gland conditions	Dry mouth	14 (1.7)	65 (0.7)
Cardiac and vascular investigations (excl enzyme tests)	Heart rate increased	5 (0.6)	34 (0.4)
	Blood pressure increased	4 (0.5)	30 (0.3)
	Heart rate irregular	1 (0.1)	4 (0)
	Blood pressure decreased	1 (0.1)	15 (0.2)
Deliria (incl confusion)	Confusional state	7 (0.8)	79 (0.9)
	Delirium	2 (0.2)	89 (1)
	Disorientation	2 (0.2)	31 (0.3)
Mood disorders and disturbances NEC	Irritability	4 (0.5)	38 (0.4)
	Mood swings	2 (0.2)	3 (0)
	Mood altered	2 (0.2)	14 (0.2)
	Frustration tolerance decreased	1 (0.1)	10 (0.1)
	Emotional distress	1 (0.1)	6 (0.1)
	Euphoric mood	1 (0.1)	10 (0.1)
Changes in physical activity	Restlessness	10 (1.2)	53 (0.6)
Lifestyle issues	Loss of personal independence in daily activities	7 (0.8)	7 (0.1)
	Impaired driving ability	2 (0.2)	8 (0.1)
	Impaired work ability	2 (0.2)	5 (0.1)
Mental impairment disorders	Disturbance in attention	5 (0.6)	24 (0.3)

	Memory impairment	3 (0.4)	73 (0.8)
	Amnesia	1 (0.1)	60 (0.7)
Off label uses and intentional product misuses/use issues	Intentional product misuse	3 (0.4)	42 (0.5)
	Off label use	3 (0.4)	38 (0.4)
	Intentional dose omission	2 (0.2)	4 (0)
	Intentional product use issue	1 (0.1)	21 (0.2)
Oral soft tissue conditions	Lip swelling	3 (0.4)	8 (0.1)
	Stomatitis	2 (0.2)	2 (0)
	Lip exfoliation	1 (0.1)	-
	Cheilitis	1 (0.1)	-
	Hypoaesthesia oral	1 (0.1)	8 (0.1)
	Oral mucosal eruption	1 (0.1)	-
	Paraesthesia oral	1 (0.1)	4 (0)
Fatal outcomes	Death	8 (0.9)	39 (0.4)
Urinary tract signs and symptoms	Nocturia	4 (0.5)	14 (0.2)
	Pollakiuria	3 (0.4)	22 (0.2)
	Urinary incontinence	1 (0.1)	6 (0.1)
Injuries NEC	Fall	4 (0.5)	101 (1.1)
	Concussion	1 (0.1)	9 (0.1)
	Scratch	1 (0.1)	6 (0.1)
	Gun shot wound	1 (0.1)	1 (0)
	Accident at home	1 (0.1)	-
Inner ear and VIIIth cranial nerve disorders	Vertigo	5 (0.6)	14 (0.2)
	Vertigo positional	1 (0.1)	-
	Tinnitus	1 (0.1)	31 (0.3)
Musculoskeletal and connective tissue disorders NEC	Pain in extremity	4 (0.5)	37 (0.4)
	Musculoskeletal stiffness	1 (0.1)	13 (0.1)
	Musculoskeletal discomfort	1 (0.1)	3 (0)

	Mobility decreased	1 (0.1)	6 (0.1)
Respiratory tract signs and symptoms	Throat tightness	3 (0.4)	8 (0.1)
	Oropharyngeal pain	1 (0.1)	6 (0.1)
	Snoring	1 (0.1)	1 (0)
	Yawning	1 (0.1)	2 (0)
	Throat irritation	1 (0.1)	2 (0)
Angioedema and urticaria	Urticaria	6 (0.7)	19 (0.2)
Eye disorders NEC	Eye swelling	2 (0.2)	3 (0)
	Dry eye	1 (0.1)	6 (0.1)
	Eye pain	1 (0.1)	10 (0.1)
	Periorbital swelling	1 (0.1)	1 (0)
	Ocular discomfort	1 (0.1)	1 (0)
Psychiatric and behavioural symptoms NEC	Hypervigilance	5 (0.6)	6 (0.1)
	Abnormal behaviour	1 (0.1)	44 (0.5)
Viral infectious disorders	COVID-19	2 (0.2)	5 (0.1)
	Influenza	2 (0.2)	11 (0.1)
	Gastroenteritis viral	1 (0.1)	-
	COVID-19 pneumonia	1 (0.1)	1 (0)
Appetite and general nutritional disorders	Decreased appetite	4 (0.5)	21 (0.2)
	Increased appetite	1 (0.1)	8 (0.1)
Cardiac arrhythmias	Tachycardia	4 (0.5)	13 (0.1)
	Arrhythmia	1 (0.1)	12 (0.1)
Infections - pathogen unspecified	Pneumonia	2 (0.2)	25 (0.3)
	Sinusitis	1 (0.1)	5 (0.1)
	Pharyngitis	1 (0.1)	1 (0)
	Oral infection	1 (0.1)	-
	Infected skin ulcer	1 (0.1)	-
	Weight increased	3 (0.4)	37 (0.4)

Physical examination and organ system status topics	Body temperature increased	2 (0.2)	1 (0)
Vision disorders	Vision blurred	2 (0.2)	16 (0.2)
	Photopsia	1 (0.1)	6 (0.1)
	Visual impairment	1 (0.1)	16 (0.2)
	Diplopia	1 (0.1)	2 (0)
Personality disorders and disturbances in behaviour	Paranoia	2 (0.2)	10 (0.1)
	Aggression	1 (0.1)	27 (0.3)
Sexual dysfunctions, disturbances and gender identity disorders	Disturbance in sexual arousal	2 (0.2)	-
	Hypersexuality	1 (0.1)	1 (0)
Upper respiratory tract disorders (excl infections)	Nasal congestion	1 (0.1)	3 (0)
	Epistaxis	1 (0.1)	7 (0.1)
	Pharyngeal swelling	1 (0.1)	-
Vascular disorders NEC	Hot flush	3 (0.4)	8 (0.1)
Cognitive and attention disorders and disturbances	Mental fatigue	1 (0.1)	1 (0)
	Daydreaming	1 (0.1)	4 (0)
Hepatic and hepatobiliary disorders	Liver disorder	1 (0.1)	8 (0.1)
	Hepatic pain	1 (0.1)	1 (0)
Seizures (incl subtypes)	Seizure	1 (0.1)	52 (0.6)
	Seizure like phenomena	1 (0.1)	3 (0)
Therapeutic procedures and supportive care NEC	Interventional procedure	1 (0.1)	-
	Therapy interrupted	1 (0.1)	-
Tongue conditions	Swollen tongue	2 (0.2)	11 (0.1)
Aural disorders NEC	Ear discomfort	1 (0.1)	1 (0)
Bone disorders (excl congenital and fractures)	Spinal disorder	1 (0.1)	3 (0)
Bronchial disorders (excl neoplasms)	Asthma	1 (0.1)	3 (0)

Central nervous system vascular disorders	Transient ischaemic attack	1 (0.1)	5 (0.1)
Cranial nerve disorders (excl neoplasms)	Parosmia	1 (0.1)	1 (0)
Cytogenetic investigations and genetic analyses	Gene mutation identification test positive	1 (0.1)	-
Decreased and nonspecific blood pressure disorders and shock	Hypotension	1 (0.1)	8 (0.1)
Dental and gingival conditions	Noninfective gingivitis	1 (0.1)	-
Gastrointestinal haemorrhages NEC	Rectal haemorrhage	1 (0.1)	2 (0)
Hearing disorders	Deafness	1 (0.1)	2 (0)
Joint disorders	Rheumatoid arthritis	1 (0.1)	-
	Joint swelling	1 (0.1)	1 (0)
	Joint stiffness	1 (0.1)	2 (0)
Metabolic, nutritional and blood gas investigations	Blood glucose increased	1 (0.1)	9 (0.1)
Middle ear disorders (excl congenital)	Middle ear effusion	1 (0.1)	(0)
Nervous system, skull and spine therapeutic procedures	Spinal operation	1 (0.1)	1 (0)
Ocular infections, irritations and inflammations	Eye pruritus	1 (0.1)	1 (0)
Ocular sensory symptoms NEC	Asthenopia	1 (0.1)	3 (0)
Prostatic disorders (excl infections and inflammations)	Prostatic disorder	1 (0.1)	2 (0)
Psychiatric disorders NEC	Mental disorder	1 (0.1)	22 (0.2)
Renal disorders (excl nephropathies)	Renal disorder	1 (0.1)	3 (0)
Skin vascular abnormalities	Skin haemorrhage	1 (0.1)	1 (0)
Toxicology and therapeutic drug monitoring	Drug screen false positive	1 (0.1)	3 (0)

Urolithiases	Nephrolithiasis	1 (0.1)	3 (0)
Vulvovaginal disorders (excl infections and inflammations)	Vaginal disorder	1 (0.1)	-
	Vulvovaginal dryness	1 (0.1)	1 (0)

[a] *ICSR: Individual Case Safety Report*

Table S3: Disproportionality analysis at Preferred Terms level for each High Level Group Term observed in daridorexant-related Individual Case Safety Reports.

High Level Group Term (HLGT)	Preferred Term (PT)	Daridorexant ICSRs[a]	Daridorexant VS RG1[b] ROR [95%CI]	Daridorexant VS RG2[c] ROR [95%CI]
Sleep disorders and disturbances	Hypnagogic hallucination	3	267.22 [85.32, 836.95]	4 [1.06, 15.1]
	Nightmare	146	113.74 [95.13, 136]	2.35 [1.93, 2.85]
	Sleep-related eating disorder	4	113.13 [42.23, 303.02]	2.67 [0.89, 7.99]
	Parasomnia	2	78.95 [19.66, 317.15]	4.26 [0.83, 22]
	Sleep terror	9	61.64 [31.93, 119.02]	1.85 [0.91, 3.77]
	Somnambulism	18	56.94 [35.67, 90.9]	1.51 [0.91, 2.48]
	Abnormal dreams	64	52.79 [40.9, 68.13]	1.16 [0.89, 1.52]
	Abnormal sleep-related event	2	33.14 [8.26, 132.9]	0.66 [0.16, 2.77]
	Sleep talking	2	27.46 [6.85, 110.11]	2.13 [0.47, 9.74]
	Middle insomnia	15	19.82 [11.89, 33.03]	0.98 [0.58, 1.68]
	Poor quality sleep	10	11.51 [6.17, 21.48]	0.55 [0.29, 1.04]
	Initial insomnia	4	9.91 [3.71, 26.46]	0.6 [0.22, 1.64]
	Insomnia	80	7.41 [5.88, 9.33]	1.94 [1.51, 2.49]
	Sleep disorder	1	0.36 [0.05, 2.57]	0.06 [0.01, 0.43]
Sleep disturbances (incl subtypes)	Sleep paralysis	21	389.34 [251.39, 602.97]	0.8 [0.51, 1.25]
	Narcolepsy	1	12.11 [1.7, 86.1]	1.18 [0.15, 9.34]
	Hypersomnia	10	8.29 [4.44, 15.47]	2.82 [1.4, 5.68]
Disturbances in thinking and perception	Autoscopy	2	111.01 [27.6, 446.44]	3.55 [0.72, 17.62]
	Tachyphrenia	5	33.02 [13.7, 79.61]	3.56 [1.29, 9.82]
	Hallucination	30	10.3 [7.15, 14.83]	1 [0.68, 1.46]
	Hallucination, visual	3	3.79 [1.22, 11.77]	1.6 [0.47, 5.39]
	Bradyphrenia	1	3.36 [0.47, 23.89]	10.65 [0.67, 170.36]
	Hallucination, auditory	2	3.06 [0.76, 12.26]	0.79 [0.19, 3.32]
	Delusion	1	1.62 [0.23, 11.54]	1.52 [0.19, 12.37]
	Thinking abnormal	1	1.16 [0.16, 8.23]	0.34 [0.05, 2.51]

Therapeutic and nontherapeutic effects (excl toxicity)	Therapeutic product effect delayed	18	46.84 [29.34, 74.75]	3.74 [2.18, 6.42]
	Therapeutic product effect variable	8	43.43 [21.63, 87.2]	8.58 [3.38, 21.8]
	Therapeutic response delayed	1	8.28 [1.16, 58.87]	0.76 [0.1, 5.78]
	Therapeutic product effect incomplete	33	7.95 [5.61, 11.26]	2.12 [1.45, 3.1]
	Drug ineffective	233	5.59 [4.81, 6.5]	0.87 [0.74, 1.02]
	Drug effect less than expected	1	4.57 [0.64, 32.5]	0.97 [0.12, 7.5]
	Therapeutic response unexpected	4	1.8 [0.67, 4.81]	1.15 [0.41, 3.24]
	Therapeutic product effect decreased	8	1.78 [0.89, 3.57]	1.15 [0.55, 2.4]
	Drug intolerance	5	1.33 [0.55, 3.21]	3.56 [1.29, 9.82]
	Drug interaction	4	0.7 [0.26, 1.88]	0.99 [0.35, 2.76]
	Treatment failure	1	0.36 [0.05, 2.54]	1.77 [0.21, 14.75]
	Drug withdrawal syndrome	1	0.23 [0.03, 1.6]	2.66 [0.3, 23.83]
Changes in physical activity	Restlessness	10	6.33 [3.4, 11.82]	2.02 [1.02, 3.98]
Middle ear disorders (excl congenital)	Middle ear effusion	1	10.84 [1.52, 77.1]	-
Product quality, supply, distribution, manufacturing and quality system issues	Product packaging difficult to open	7	104.91 [49.76, 221.2]	5.35 [2.15, 13.3]
	Product availability issue	51	97.47 [73.39, 129.45]	32 [18.61, 55.04]
	Product after taste	1	55.44 [7.78, 395.19]	-
	Product container seal issue	2	41.78 [10.42, 167.6]	-
	Product shape issue	1	24.03 [3.38, 170.98]	5.32 [0.48, 58.76]
	Product container issue	2	6.21 [1.55, 24.87]	7.1 [1.19, 42.58]
	Product colour issue	1	5.35 [0.75, 38.02]	1.77 [0.21, 14.75]
	Product taste abnormal	2	3.54 [0.88, 14.19]	10.66 [1.5, 75.76]
	Product packaging issue	1	2.7 [0.38, 19.19]	1.06 [0.14, 8.32]
	Product physical issue	2	2.43 [0.61, 9.74]	7.1 [1.19, 42.58]
	Product quality issue	1	0.15 [0.02, 1.08]	0.16 [0.02, 1.12]
Salivary gland conditions	Dry mouth	14	4.17 [2.46, 7.07]	2.31 [1.29, 4.14]
Headaches	Migraine with aura	2	16.56 [4.13, 66.38]	21.32 [1.93, 235.34]
	Headache	59	2.27 [1.74, 2.95]	1.52 [1.15, 2.01]
	Migraine	8	2.05 [1.02, 4.12]	2.25 [1.05, 4.84]
	Mental fatigue	1	20.68 [2.91, 147.15]	10.65 [0.67, 170.36]

Cognitive and attention disorders and disturbances	Daydreaming	1	14.73 [2.07, 104.75]	2.66 [0.3, 23.83]
Sexual dysfunctions, disturbances and gender identity disorders	Disturbance in sexual arousal	2	50.12 [12.49, 201.1]	-
	Hypersexuality	1	14.23 [2, 101.22]	10.65 [0.67, 170.36]
Psychiatric and behavioural symptoms NEC	Hypervigilance	5	124.4 [51.49, 300.53]	8.91 [2.71, 29.25]
	Abnormal behaviour	1	0.53 [0.08, 3.8]	0.23 [0.03, 1.67]
Anxiety disorders and symptoms	Phobia of driving	1	145.1 [20.26, 1039.18]	10.65 [0.67, 170.36]
	Agitation	16	5.06 [3.09, 8.3]	1.72 [1.01, 2.92]
	Nervousness	5	2.02 [0.84, 4.87]	1.24 [0.49, 3.13]
	Panic attack	3	1.88 [0.6, 5.83]	1.1 [0.33, 3.62]
	Anxiety	14	1.1 [0.65, 1.86]	0.72 [0.42, 1.24]
	Fear	1	0.76 [0.11, 5.4]	0.25 [0.03, 1.84]
	Stress	1	0.33 [0.05, 2.32]	0.63 [0.08, 4.7]
Depressed mood disorders and disturbances	Depression suicidal	2	13.3 [3.32, 53.31]	21.32 [1.93, 235.34]
	Depression	22	2.13 [1.39, 3.25]	2.31 [1.45, 3.67]
	Depressed mood	3	1.36 [0.44, 4.24]	1.45 [0.43, 4.86]
Suicidal and self-injurious behaviours NEC	Suicidal ideation	13	3.3 [1.91, 5.7]	1.7 [0.94, 3.06]
	Suicide attempt	2	0.84 [0.21, 3.38]	0.47 [0.11, 1.95]
	Completed suicide	1	0.37 [0.05, 2.63]	0.29 [0.04, 2.09]
Neurological disorders NEC	Restless legs syndrome	7	8.81 [4.19, 18.54]	2.14 [0.95, 4.82]
	Somnolence	55	6.88 [5.23, 9.04]	0.95 [0.71, 1.26]
	Sedation	5	5.18 [2.15, 12.47]	1.37 [0.54, 3.47]
	Lethargy	8	3.27 [1.63, 6.57]	3.43 [1.54, 7.62]
	Myoclonus	1	2.36 [0.33, 16.75]	1.52 [0.19, 12.37]
	Head discomfort	1	1.41 [0.2, 10]	0.41 [0.06, 3.01]
	Dizziness	29	1.36 [0.94, 1.97]	1.45 [0.98, 2.15]
	Neuralgia	1	0.99 [0.14, 7.06]	-
	Ageusia	1	0.91 [0.13, 6.5]	5.32 [0.48, 58.76]
	Hypoaesthesia	6	0.9 [0.4, 2.02]	1.83 [0.77, 4.36]
	Speech disorder	2	0.87 [0.22, 3.47]	0.79 [0.19, 3.32]
	Aphasia	1	0.75 [0.11, 5.36]	0.71 [0.09, 5.37]

	Dysgeusia	2	0.58 [0.15, 2.34]	0.89 [0.21, 3.76]
	Paraesthesia	4	0.56 [0.21, 1.51]	1.22 [0.43, 3.43]
	Burning sensation	1	0.32 [0.04, 2.27]	1.06 [0.14, 8.32]
	Balance disorder	1	0.26 [0.04, 1.84]	0.46 [0.06, 3.42]
	Loss of consciousness	1	0.18 [0.03, 1.28]	0.22 [0.03, 1.57]
Movement disorders (incl parkinsonism)	Resting tremor	1	51.02 [7.16, 363.62]	-
	Freezing phenomenon	1	7.91 [1.11, 56.26]	-
	Bradykinesia	1	5.04 [0.71, 35.81]	-
	Psychomotor hyperactivity	3	4.04 [1.3, 12.55]	1.18 [0.36, 3.91]
	Dyskinesia	5	2.88 [1.2, 6.94]	3.14 [1.16, 8.53]
	Tremor	15	2.07 [1.24, 3.45]	2.15 [1.23, 3.76]
	Paralysis	1	1.6 [0.23, 11.39]	0.25 [0.03, 1.79]
Inner ear and VIIIth cranial nerve disorders	Vertigo positional	1	13.24 [1.86, 94.14]	-
	Vertigo	5	1.86 [0.77, 4.49]	3.81 [1.37, 10.62]
	Tinnitus	1	0.51 [0.07, 3.61]	0.32 [0.04, 2.35]
Cardiac disorders, signs and symptoms NEC	Cardiac discomfort	1	12.15 [1.71, 86.44]	-
	Palpitations	15	3.01 [1.8, 5.01]	1.61 [0.93, 2.78]
Overdoses and underdoses NEC	Intentional overdose	17	7.73 [4.78, 12.5]	2.82 [1.64, 4.83]
	Prescribed overdose	3	3.77 [1.21, 11.72]	1.52 [0.45, 5.11]
	Prescribed underdose	1	1.25 [0.18, 8.85]	1.52 [0.19, 12.37]
	Overdose	3	0.34 [0.11, 1.05]	0.23 [0.07, 0.72]
Allergic conditions	Hypersensitivity	29	3.78 [2.61, 5.47]	24.53 [12.7, 47.37]
	Drug hypersensitivity	1	0.12 [0.02, 0.82]	0.44 [0.06, 3.27]
Prostatic disorders (excl infections and inflammations)	Prostatic disorder	1	3.85 [0.54, 27.36]	5.32 [0.48, 58.76]
General system disorders NEC	Hangover	20	127.92 [81.98, 199.62]	3.38 [2.04, 5.61]
	Energy increased	5	18.7 [7.76, 45.06]	6.68 [2.18, 20.47]
	Concomitant disease aggravated	4	15.43 [5.78, 41.23]	-
	Screaming	3	10.74 [3.46, 33.39]	2.66 [0.75, 9.46]
	Sluggishness	4	9.02 [3.38, 24.09]	3.05 [1, 9.28]
	Illness	17	7.19 [4.44, 11.62]	18.43 [8.41, 40.38]

	Feeling jittery	6	6.89 [3.08, 15.37]	1.25 [0.54, 2.93]
	Feeling abnormal	64	6.19 [4.8, 7.99]	1.75 [1.33, 2.29]
	Performance status decreased	1	5.04 [0.71, 35.83]	-
	Localised oedema	1	4.89 [0.69, 34.75]	-
	Crying	5	2.95 [1.22, 7.1]	2.67 [1, 7.13]
	Feeling drunk	1	2.93 [0.41, 20.83]	0.59 [0.08, 4.43]
	Feeling cold	2	1.63 [0.41, 6.52]	3.04 [0.63, 14.67]
	Fatigue	45	1.41 [1.05, 1.91]	2.28 [1.64, 3.17]
	Face oedema	1	1.36 [0.19, 9.7]	10.65 [0.67, 170.36]
	Discomfort	3	1.16 [0.37, 3.6]	2.91 [0.81, 10.44]
	Swelling face	3	1.06 [0.34, 3.3]	2.91 [0.81, 10.44]
	Gait inability	2	0.81 [0.2, 3.24]	5.33 [0.97, 29.13]
	Malaise	12	0.61 [0.34, 1.07]	1.07 [0.59, 1.95]
	Chest pain	4	0.48 [0.18, 1.28]	1.29 [0.46, 3.65]
	Chest discomfort	2	0.46 [0.12, 1.86]	0.92 [0.22, 3.93]
	Chills	2	0.39 [0.1, 1.57]	1.12 [0.26, 4.82]
	Asthenia	6	0.37 [0.17, 0.83]	1.16 [0.5, 2.71]
	Peripheral swelling	2	0.32 [0.08, 1.29]	3.04 [0.63, 14.67]
	Condition aggravated	3	0.27 [0.09, 0.83]	1.88 [0.55, 6.43]
	Influenza like illness	1	0.26 [0.04, 1.86]	1.52 [0.19, 12.37]
	Gait disturbance	2	0.23 [0.06, 0.92]	0.57 [0.14, 2.39]
	Swelling	1	0.2 [0.03, 1.46]	3.55 [0.37, 34.15]
	Pain	4	0.14 [0.05, 0.38]	0.82 [0.29, 2.26]
Lifestyle issues	Impaired driving ability	2	3.94 [0.98, 15.77]	2.66 [0.56, 12.56]
	Loss of personal independence in daily activities	7	2.47 [1.17, 5.19]	10.71 [3.75, 30.62]
	Impaired work ability	2	2.04 [0.51, 8.16]	4.26 [0.83, 22]
Deliria (incl confusion)	Delirium	2	1.63 [0.41, 6.54]	0.23 [0.06, 0.95]
	Disorientation	2	1.13 [0.28, 4.52]	0.66 [0.16, 2.77]
	Confusional state	7	1.01 [0.48, 2.12]	0.93 [0.43, 2.02]
	Intercepted product prescribing error	1	15.59 [2.19, 110.92]	10.65 [0.67, 170.36]

Medication errors and other product use errors and issues	Product prescribing issue	4	9.53 [3.57, 25.46]	3.28 [1.07, 10.09]
	Intercepted product dispensing error	1	8.41 [1.18, 59.8]	10.65 [0.67, 170.36]
	Inappropriate schedule of product administration	48	5.47 [4.08, 7.32]	6.31 [4.39, 9.05]
	Product prescribing error	8	4.36 [2.18, 8.76]	5.72 [2.42, 13.52]
	Wrong technique in product usage process	35	3.7 [2.64, 5.19]	1.78 [1.24, 2.56]
	Product dispensing error	3	1.96 [0.63, 6.1]	8 [1.79, 35.81]
	Contraindicated product administered	2	1.72 [0.43, 6.9]	2.37 [0.51, 10.97]
	Product use issue	4	0.55 [0.21, 1.48]	0.37 [0.14, 1]
	Product storage error	1	0.32 [0.04, 2.24]	0.48 [0.06, 3.59]
	Incorrect dose administered	1	0.13 [0.02, 0.89]	0.26 [0.04, 1.88]
Nervous system, skull and spine therapeutic procedures	Spinal operation	1	2.45 [0.34, 17.43]	10.65 [0.67, 170.36]
Oral soft tissue conditions	Lip exfoliation	1	17.74 [2.49, 126.19]	-
	Oral mucosal eruption	1	15.95 [2.24, 113.49]	-
	Cheilitis	1	4.2 [0.59, 29.88]	-
	Lip swelling	3	2.1 [0.68, 6.53]	4 [1.06, 15.1]
	Paraesthesia oral	1	1.66 [0.23, 11.8]	2.66 [0.3, 23.83]
	Hypoaesthesia oral	1	1.51 [0.21, 10.74]	1.33 [0.17, 10.64]
	Stomatitis	2	0.83 [0.21, 3.31]	10.66 [1.5, 75.76]
Skin appendage conditions	Night sweats	11	8.48 [4.68, 15.38]	3.94 [1.97, 7.89]
	Hyperhidrosis	8	1.41 [0.7, 2.82]	1.78 [0.84, 3.77]
	Alopecia	2	0.23 [0.06, 0.92]	10.66 [1.5, 75.76]
Cytogenetic investigations and genetic analyses	Gene mutation identification test positive	1	58.4 [8.19, 416.38]	-
Ocular sensory symptoms NEC	Asthenopia	1	4.44 [0.62, 31.57]	3.55 [0.37, 34.15]
Eye disorders NEC	Periorbital swelling	1	6.98 [0.98, 49.62]	10.65 [0.67, 170.36]
	Ocular discomfort	1	2.74 [0.39, 19.49]	10.65 [0.67, 170.36]
	Eye swelling	2	1.27 [0.32, 5.11]	7.1 [1.19, 42.58]
	Dry eye	1	0.59 [0.08, 4.2]	1.77 [0.21, 14.75]
	Eye pain	1	0.47 [0.07, 3.34]	1.06 [0.14, 8.32]

Urinary tract signs and symptoms	Nocturia	4	8.13 [3.04, 21.72]	3.05 [1, 9.28]
	Pollakiuria	3	1.66 [0.53, 5.16]	1.45 [0.43, 4.86]
	Urinary incontinence	1	0.78 [0.11, 5.52]	1.77 [0.21, 14.75]
Mood disorders and disturbances NEC	Euphoric mood	1	2.08 [0.29, 14.79]	1.06 [0.14, 8.32]
	Frustration tolerance decreased	1	1.96 [0.28, 13.94]	1.06 [0.14, 8.32]
	Mood altered	2	1.71 [0.43, 6.86]	1.52 [0.34, 6.7]
	Irritability	4	1.49 [0.56, 3.97]	1.06 [0.38, 2.98]
	Mood swings	2	1.36 [0.34, 5.45]	7.1 [1.19, 42.58]
	Emotional distress	1	0.16 [0.02, 1.16]	1.77 [0.21, 14.75]
Angioedema and urticaria	Urticaria	6	0.88 [0.39, 1.96]	3.38 [1.34, 8.47]
Personality disorders and disturbances in behaviour	Paranoia	2	2.62 [0.65, 10.48]	2.13 [0.47, 9.74]
	Aggression	1	0.45 [0.06, 3.18]	0.39 [0.05, 2.9]
Mental impairment disorders	Disturbance in attention	5	2.11 [0.87, 5.07]	2.22 [0.85, 5.84]
	Memory impairment	3	0.5 [0.16, 1.55]	0.42 [0.13, 1.35]
	Amnesia	1	0.34 [0.05, 2.39]	0.17 [0.02, 1.21]
Tongue conditions	Swollen tongue	2	1.43 [0.36, 5.72]	1.94 [0.43, 8.75]
Muscle disorders	Muscle twitching	3	2.98 [0.96, 9.27]	2.66 [0.75, 9.46]
	Muscle rigidity	1	2.12 [0.3, 15.05]	2.13 [0.25, 18.24]
	Muscular weakness	3	0.62 [0.2, 1.92]	0.59 [0.18, 1.89]
	Muscle spasms	5	0.61 [0.25, 1.47]	1.57 [0.61, 4.02]
	Myalgia	3	0.41 [0.13, 1.27]	1 [0.3, 3.26]
Cardiac and vascular investigations (excl enzyme tests)	Heart rate increased	5	1.18 [0.49, 2.84]	1.57 [0.61, 4.02]
	Heart rate irregular	1	0.94 [0.13, 6.69]	2.66 [0.3, 23.83]
	Blood pressure increased	4	0.6 [0.23, 1.61]	1.42 [0.5, 4.04]
	Blood pressure decreased	1	0.35 [0.05, 2.49]	0.71 [0.09, 5.37]
Gastrointestinal signs and symptoms	Abdominal discomfort	8	1.1 [0.55, 2.21]	1.99 [0.93, 4.24]
	Nausea	23	0.68 [0.45, 1.03]	1.24 [0.8, 1.91]
	Abdominal pain upper	5	0.57 [0.24, 1.38]	2.22 [0.85, 5.84]
	Vomiting	7	0.35 [0.17, 0.75]	1.08 [0.49, 2.36]
	Dysphagia	1	0.24 [0.03, 1.7]	0.48 [0.06, 3.59]
	Dyspepsia	1	0.24 [0.03, 1.7]	0.59 [0.08, 4.43]

	Abdominal distension	1	0.23 [0.03, 1.63]	1.52 [0.19, 12.37]
	Abdominal pain	1	0.1 [0.01, 0.72]	0.53 [0.07, 3.96]
Aural disorders NEC	Ear discomfort	1	2.31 [0.33, 16.44]	10.65 [0.67, 170.36]
Gastrointestinal motility and defaecation conditions	Diarrhoea haemorrhagic	1	2.54 [0.36, 18.03]	-
	Irritable bowel syndrome	1	1.16 [0.16, 8.24]	-
	Diarrhoea	15	0.57 [0.34, 0.94]	1.64 [0.95, 2.84]
	Constipation	4	0.46 [0.17, 1.22]	1.85 [0.64, 5.37]
	Gastrooesophageal reflux disease	1	0.28 [0.04, 2.02]	0.82 [0.11, 6.26]
Epidermal and dermal conditions	Skin odour abnormal	1	5.75 [0.81, 40.88]	10.65 [0.67, 170.36]
	Dermatitis contact	1	2.16 [0.3, 15.33]	-
	Rash papular	1	0.99 [0.14, 7.06]	10.65 [0.67, 170.36]
	Pruritus	15	0.97 [0.58, 1.62]	2.52 [1.43, 4.44]
	Rash	15	0.79 [0.47, 1.32]	2.34 [1.33, 4.1]
	Rash erythematous	1	0.58 [0.08, 4.1]	2.66 [0.3, 23.83]
	Skin irritation	1	0.48 [0.07, 3.41]	-
	Erythema	4	0.44 [0.16, 1.17]	3.05 [1, 9.28]
	Psoriasis	1	0.18 [0.02, 1.25]	5.32 [0.48, 58.76]
Urolithiases	Nephrolithiasis	1	0.53 [0.07, 3.76]	3.55 [0.37, 34.15]
Respiratory disorders NEC	Respiratory tract congestion	1	1.5 [0.21, 10.68]	-
	Dyspnoea	20	0.82 [0.53, 1.28]	2.11 [1.3, 3.43]
	Cough	1	0.09 [0.01, 0.61]	0.38 [0.05, 2.79]
Skin vascular abnormalities	Skin haemorrhage	1	2.01 [0.28, 14.32]	10.65 [0.67, 170.36]
Respiratory tract signs and symptoms	Yawning	1	8.43 [1.19, 59.94]	5.32 [0.48, 58.76]
	Snoring	1	6.2 [0.87, 44.08]	10.65 [0.67, 170.36]
	Throat tightness	3	2.55 [0.82, 7.93]	4 [1.06, 15.1]
	Throat irritation	1	0.54 [0.08, 3.8]	5.32 [0.48, 58.76]
	Oropharyngeal pain	1	0.25 [0.04, 1.81]	1.77 [0.21, 14.75]
Cranial nerve disorders (excl neoplasms)	Parosmia	1	3.1 [0.44, 22.02]	10.65 [0.67, 170.36]
Toxicology and therapeutic drug monitoring	Drug screen false positive	1	7.49 [1.05, 53.26]	3.55 [0.37, 34.15]

Appetite and general nutritional disorders	Increased appetite	1	1.42 [0.2, 10.1]	1.33 [0.17, 10.64]
	Decreased appetite	4	0.39 [0.15, 1.05]	2.03 [0.7, 5.93]
Vulvovaginal disorders (excl infections and inflammations)	Vaginal disorder	1	22.57 [3.17, 160.58]	-
	Vulvovaginal dryness	1	7.13 [1, 50.72]	10.65 [0.67, 170.36]
Upper respiratory tract disorders (excl infections)	Pharyngeal swelling	1	3.3 [0.46, 23.44]	-
	Nasal congestion	1	0.42 [0.06, 3.01]	3.55 [0.37, 34.15]
	Epistaxis	1	0.31 [0.04, 2.19]	1.52 [0.19, 12.37]
Viral infectious disorders	COVID-19 pneumonia	1	2.61 [0.37, 18.57]	10.65 [0.67, 170.36]
	Gastroenteritis viral	1	1.36 [0.19, 9.65]	-
	Influenza	2	0.45 [0.11, 1.81]	1.94 [0.43, 8.75]
	COVID-19	2	0.34 [0.08, 1.34]	2.66 [0.56, 12.56]
Hearing disorders	Deafness	1	0.93 [0.13, 6.63]	5.32 [0.48, 58.76]
Vascular disorders NEC	Hot flush	3	1 [0.32, 3.11]	4 [1.06, 15.1]
Off label uses and intentional product misuses/use issues	Intentional dose omission	2	3.45 [0.86, 13.81]	4.26 [0.83, 22]
	Intentional product misuse	3	0.64 [0.2, 1.97]	0.76 [0.23, 2.45]
	Intentional product use issue	1	0.32 [0.04, 2.26]	0.51 [0.07, 3.77]
	Off label use	3	0.11 [0.03, 0.33]	0.78 [0.24, 2.52]
Vision disorders	Photopsia	1	3.57 [0.5, 25.41]	1.77 [0.21, 14.75]
	Diplopia	1	0.94 [0.13, 6.65]	5.32 [0.48, 58.76]
	Vision blurred	2	0.34 [0.09, 1.38]	1.33 [0.31, 5.79]
	Visual impairment	1	0.19 [0.03, 1.34]	0.66 [0.09, 5.02]
Cardiac arrhythmias	Tachycardia	4	1.13 [0.42, 3.01]	3.28 [1.07, 10.09]
	Arrhythmia	1	0.48 [0.07, 3.44]	0.82 [0.11, 6.26]
Fatal outcomes	Death	8	0.22 [0.11, 0.44]	2.04 [0.95, 4.35]
Dental and gingival conditions	Noninfective gingivitis	1	16.11 [2.26, 114.58]	-
Injuries NEC	Accident at home	1	45.52 [6.39, 324.31]	-
	Gun shot wound	1	10.83 [1.52, 77.01]	10.65 [0.67, 170.36]
	Concussion	1	3.05 [0.43, 21.66]	0.97 [0.12, 7.5]
	Scratch	1	2.71 [0.38, 19.27]	1.77 [0.21, 14.75]
	Fall	4	0.28 [0.1, 0.74]	0.41 [0.15, 1.12]
	Body temperature increased	2	2.2 [0.55, 8.81]	21.32 [1.93, 235.34]

Physical examination and organ system status topics	Weight increased	3	0.32 [0.1, 0.98]	0.86 [0.27, 2.8]
Musculoskeletal and connective tissue disorders NEC	Musculoskeletal discomfort	1	1.35 [0.19, 9.59]	3.55 [0.37, 34.15]
	Mobility decreased	1	0.33 [0.05, 2.35]	1.77 [0.21, 14.75]
	Pain in extremity	4	0.3 [0.11, 0.8]	1.12 [0.4, 3.15]
	Musculoskeletal stiffness	1	0.26 [0.04, 1.88]	0.82 [0.11, 6.26]
Seizures (incl subtypes)	Seizure like phenomena	1	16.51 [2.32, 117.43]	3.55 [0.37, 34.15]
	Seizure	1	0.14 [0.02, 0.98]	0.2 [0.03, 1.47]
Hepatic and hepatobiliary disorders	Hepatic pain	1	5.36 [0.75, 38.11]	10.65 [0.67, 170.36]
	Liver disorder	1	0.55 [0.08, 3.92]	1.33 [0.17, 10.64]
Ocular infections, irritations and inflammations	Eye pruritus	1	0.85 [0.12, 6.06]	10.65 [0.67, 170.36]
Therapeutic procedures and supportive care NEC	Interventional procedure	1	357.16 [49.32, 2586.57]	-
	Therapy interrupted	1	0.64 [0.09, 4.54]	-
Bronchial disorders (excl neoplasms)	Asthma	1	0.24 [0.03, 1.68]	3.55 [0.37, 34.15]
Bone disorders (excl congenital and fractures)	Spinal disorder	1	2.37 [0.33, 16.88]	3.55 [0.37, 34.15]
Gastrointestinal haemorrhages NEC	Rectal haemorrhage	1	0.52 [0.07, 3.71]	5.32 [0.48, 58.76]
Decreased and nonspecific blood pressure disorders and shock	Hypotension	1	0.12 [0.02, 0.88]	1.33 [0.17, 10.64]
Infections - pathogen unspecified	Infected skin ulcer	1	11.81 [1.66, 83.96]	-
	Oral infection	1	5.8 [0.82, 41.24]	-
	Pharyngitis	1	1.82 [0.26, 12.94]	10.65 [0.67, 170.36]
	Sinusitis	1	0.23 [0.03, 1.61]	2.13 [0.25, 18.24]
	Pneumonia	2	0.14 [0.03, 0.56]	0.82 [0.19, 3.45]
Psychiatric disorders NEC	Mental disorder	1	0.53 [0.08, 3.79]	0.48 [0.06, 3.59]
Central nervous system vascular disorders	Transient ischaemic attack	1	0.65 [0.09, 4.6]	1.77 [0.21, 14.75]
Metabolic, nutritional and blood gas investigations	Blood glucose increased	1	0.12 [0.02, 0.82]	1.18 [0.15, 9.34]
Renal disorders (excl nephropathies)	Renal disorder	1	0.5 [0.07, 3.54]	3.55 [0.37, 34.15]

Joint disorders	Joint stiffness	1	0.87 [0.12, 6.21]	5.32 [0.48, 58.76]
	Rheumatoid arthritis	1	0.21 [0.03, 1.49]	-
	Joint swelling	1	0.2 [0.03, 1.42]	10.65 [0.67, 170.36]

[a]*ICSR; Individual Case Safety Report*

[b]*RG1; Reference Group 1, report relative to all other drugs in the FAERS database*

[c]*RG2; Reference Group 2, report relative to other DORAs*
